# Conformational Dynamics and Ligand Binding in the Multi-Domain Protein PDC109

**DOI:** 10.1371/journal.pone.0009180

**Published:** 2010-02-18

**Authors:** Hyun Jin Kim, Moo Young Choi, Hyung J. Kim, Miguel Llinás

**Affiliations:** 1 Department of Chemistry, Carnegie Mellon University, Pittsburgh, Pennsylvania, United States of America; 2 Department of Physics and Center for Theoretical Physics, Seoul National University, Seoul, Korea; 3 School of Computational Sciences, Korea Institute for Advanced Study, Seoul, Korea; Massachusetts Institute of Technology, United States of America

## Abstract

PDC109 is a modular multi-domain protein with two fibronectin type II (Fn2) repeats joined by a linker. It plays a major role in bull sperm binding to the oviductal epithelium through its interactions with phosphorylcholines (PhCs), a head group of sperm cell membrane lipids. The crystal structure of the PDC109-PhC complex shows that each PhC binds to the corresponding Fn2 domain, while the two domains are on the same face of the protein. Long timescale explicit solvent molecular dynamics (MD) simulations of PDC109, in the presence and absence of PhC, suggest that PhC binding strongly correlates with the relative orientation of choline-phospholipid binding sites of the two Fn2 domains; unless the two domains tightly bind PhCs, they tend to change their relative orientation by deforming the flexible linker. The effective PDC109-PhC association constant of 28 M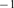, estimated from their potential of mean force is consistent with the experimental result. Principal component analysis of the long timescale MD simulations was compared to the significantly less expensive normal mode analysis of minimized structures. The comparison indicates that difference between relative domain motions of PDC109 with bound and unbound PhC is captured by the first principal component in the principal component analysis as well as the three lowest normal modes in the normal mode analysis. The present study illustrates the use of detailed MD simulations to clarify the energetics of specific ligand-domain interactions revealed by a static crystallographic model, as well as their influence on relative domain motions in a multi-domain protein.

## Introduction

Biological function of macromolecules depends both on their structures folds and on their dynamic characteristics, i.e., the “conformational state” [1]. Single domain proteins (or individual domains within multi-domain proteins) can exhibit essentially identical structures, yet measurably differ in their global plasticity and function with even a single amino acid mutation [2]. Characterization of conformational states of multi-domain proteins additionally requires description of inter-domain relative motions that can be influenced by long-range interactions. The overall supra-fold dynamics of multi-domain proteins is thus governed by time dependent changes in both the internal conformations of individual domains and the 3D organization of these domains with respect to each other. This supra-fold dynamics can, in principle, be altered by ligand binding to protein domains, which can change both individual protein domain structures as well as their relative arrangement [3], [4]. Characterizing ligand binding and its influence on the detailed supra-fold dynamics of multi-domain proteins therefore requires atomic resolution techniques.

The structural details of protein-ligand interactions can be investigated both experimentally [4]–[9] and through predictive docking calculations [10]–[13]. Traditional computational docking methods employ rather accurate force fields but commonly do not comprehensively explore dynamic adaptation of protein conformations to induce ligand binding [14]–[17]. Solvent effects are also usually ignored or treated through less accurate implicit solvent models [18]. Explicit solvent MD simulations can circumvent these limitations of traditional docking methods by treating protein and solvent response to ligand binding explicitly. They are also suited to identify potential higher energy state conformations that are not easily accessible in experimental studies. Owing to the availability of massively parallel computational resources, such MD simulations are now feasible to investigate intra- and inter-domain conformational dynamics in multi-domain proteins in atomic detail. Computational studies using explicit solvent MD simulations have been successfully employed to probe ligand binding with individual protein domains [19]–[23]. However, little is known on the effects of ligand binding on the supra-fold dynamics of multi-domain proteins.

PDC109 is a 10.6 kDa modular, two-domain protein that induces sperm capacitation by interacting with sperm cell membranes [25]. The interaction of PDC109 with spermatozoa stimulates the efflux of phosphorylcholine (PhC), the soluble head group of the sperm cell membranes, resulting in the specific binding to PDC109 domains [24], [31], [38]. This appears to be an important step in the capacitation process, before fertilization can occur. It follows that in order to characterize the molecular events involved in the capacitation process, the binding mechanism between PDC109 and PhC should be better understood.

**Figure 1:**
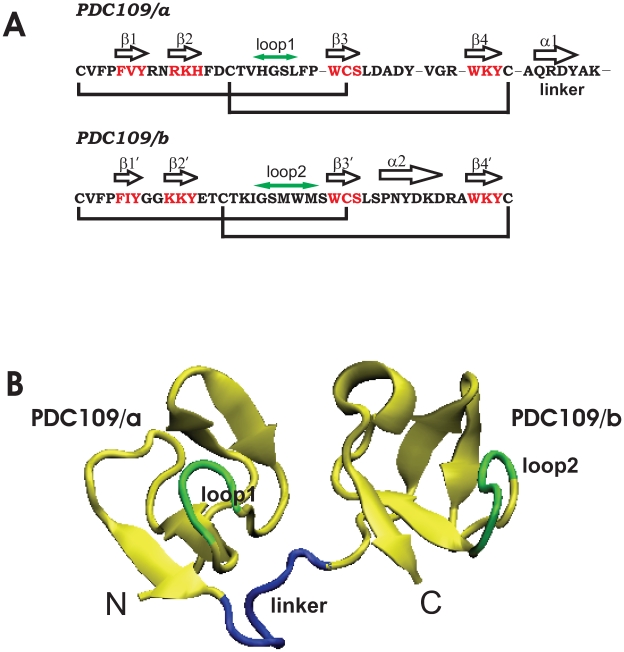
X-ray structure of BSP-A1. (A) Sequence and associated secondary structure organization of PDC109. Cystine bridges are indicated by black lines. (B) Crystal structure of PDC109 [24]. The N-terminal Fn2 domain (PDC109/a, residues 24–61) and the C-terminal Fn2 domain (PDC109/b, residues 69–109) are connected by a linker peptide (residues 62–68) shown in blue. The net charges are 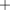
1, 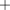
1, and 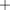
2 for PDC109/a, linker, and PDC109/b, respectively. Loop 1 (H41-L44) between 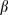
2 and 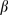
3 strands in PDC109/a and loop 2 (G87-M91) between the 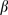
2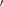 and 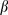
3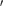 strands in PDC109/b are denoted by green arrows [74].

**Figure 2:**
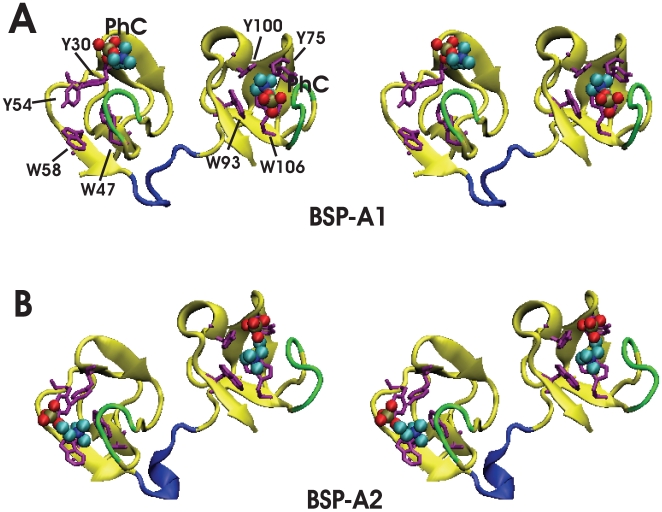
Stereoviews of the homodimer crystal structure of PDC109 complexed with PhCs (PDB ID: 1h8p) [24]. (A) BSP-A1 protomer and (B) BSP-A2 protomer. Bound PhC molecules are shown as vdW spheres, aromatic sidechains at the PhC binding sites are shown in orange, and loops 1 and 2 that neighbor the binding sites and interact with PhC ligand are denoted in green [74].

The structure of the complex of PDC109 with PhC [24] has been solved as a homodimer using X-ray crystallography (Figs. 1 and 2). The dimer is composed of two protomers, BSP-A1 and BSP-A2, which only differ in the extent of glycosylation [26], [27]. PDC109 refers to a mixture of BSP-A1 and BSP-A2. It is composed of 109 amino acids with an N-terminal O-glycosylated acidic extension followed by two fibronectin type II (Fn2) repeats, where each has the capability to bind to one PhC molecule [24]. For the sake of simplicity, the N-terminal (residues 24–61) and C-terminal (residues 69–109) Fn2 domains of PDC109 are denoted as PDC109/a and PDC109/b, respectively.

The crystal structure shows that the N-terminal glycosylated tails are disordered and that PhC is slightly displaced from its PDC109/a binding site in BSP-A1 (Fig. 2). The solution structure of PDC109/b has been determined by NMR spectroscopy and has been shown to also bind collagen in a specific binding pocket [28].

The interaction of PDC109 with phospholipid membranes takes place via specific interaction of PhC with the protein [26], [27], [29]–[46]. The reported experimental characterization of ligand binding properties as well as the small size and the multi-domain organization and structural simplicity of PDC109 qualify it as an ideal model system to explore the use of explicit solvent MD simulations to study ligand binding and conformational dynamics in multi-domain proteins. These studies motivate us to undertake a characterization of the effect of PhC binding on the molecular dynamics (MD) properties of PDC109.

Large-scale structural reorganizations involved in the biological functions of proteins can also be computationally analyzed using either normal mode analysis (NMA) or principal components analysis (PCA) [47]. In NMA, the assumption is that atomic positions in the equilibrated structure are governed by harmonic potentials. Within this approximation, eigenvectors obtained via diagonalization of the Hessian matrix of derivatives of atomic forces in minimum energy structures provide useful insights into global dynamics of proteins. In contrast, PCA is obtained from statistical analysis of MD trajectories and captures non-harmonic effects stemming from the protein energy landscape and solvent dynamics.

In the present study, we utilize long timescale explicit solvent MD simulations to analyze differences between the ligand-free and ligand-bound states of PDC109 as well as the effect of PhC ligand binding on the domain-domain interactions. Effects of PhC binding are identified both with respect to the local dynamics around binding sites and the relative motion of the two PhC binding domains. We show that the MD simulation results are validated by estimating the association constant (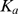) of PhC to PDC109 via potentials of mean force. We find that the calculated constant matches well with the experimental result. We also apply PCA to the detailed MD simulation trajectories and compare it to the simplified NMA on minimized structures in order to identify effective strategies for exploring multi-domain protein motions in a computationally affordable fashion.

## Results and Discussion

### Effects of PhC Binding on Intra-Domain Dynamics

#### Ligand binding geometries

Direct evidence for the specificity of PDC109 towards PhC moiety came from the crystal X-ray diffraction study [24]. Based on the crystal structure, it has been proposed that PhC interacts with the Fn2 domains through cation-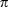 interactions [48]–[51] between the quaternary ammonium group and binding site tryptophan residues W47 and W58 (PDC109/a) and W93 and W106 (PDC109/b) (Fig. 1 A and B). Hydrogen bonding was also postulated to occur between the PhC phosphate and exposed hydroxyl groups of tyrosine residues Y30 and Y54 (PDC109/a) and Y75 and Y100 (PDC109/b).

**Figure 3:**
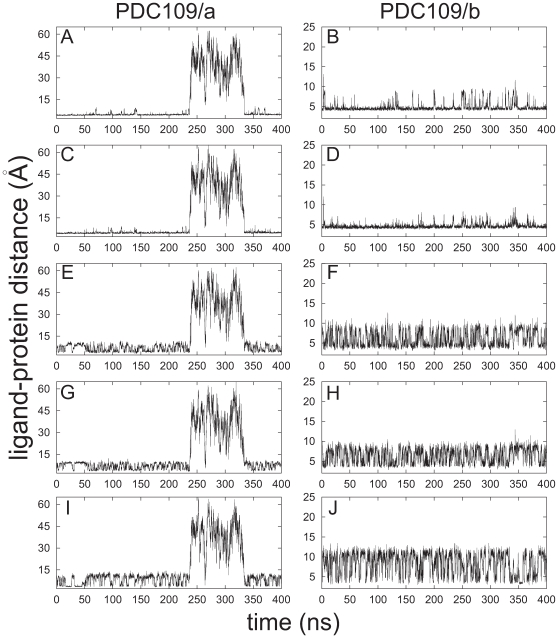
Distances between the ligand and protein interaction sites in PDC109 domains. Time series are shown for distances between the quaternary ammonium nitrogen of PhC and the center of geometry of six carbon atoms in indole rings of W47 (A), W93 (B), W58 (C), W106 (D); and between the average position of anionic PhC phosphoryl oxygens and the hydroxyl oxygens of Y30 (E), Y75 (F), Y54 (G), Y100 (H), Y60 (I), Y108 (J).

Time series of distance profiles monitoring PhC ligand binding during the MD simulations for the PDC109-PhC complex are shown in Fig. 3. The pair-wise atomic distances used to monitor ligand binding geometries are either between the PhC quaternary ammonium nitrogen and specific binding site tryptophan residue side-chains (W47, W58 from PDC109/a and W93, W106 from PDC109/b) or between the PhC phosphate anionic oxygens and specific binding site tyrosine residues (Y30, Y54, Y60 from PDC109/a and Y75, Y100, Y108 from PDC109/b). On the basis of a quantum mechanical potential, a minimum cation-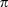 distance is estimated at around 3.0 Å [48]. As illustrated in Fig. 3 A–D, in the MD simulations, tryptophan-PhC distances mostly remain around 4.5 Å, with sporadic increases. The energy function used in the MD simulations does not include an explicit cation-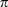 interaction. However, the observed average value around 4.5 Å is close to the value of 4.4 Å, obtained by averaging over PDC109/b in protomer A, PDC109/a in protomer B, and PDC109/b in protomer B in the crystal structure [24]. Because the anomalous ligand position in PDC109/a of protomer A in the crystal structure [24], the PDC109/a-PhC complex was excluded from the average (Fig. 1 A). This unbound state in the crystal structure was also observed during the MD simulations. The PhC molecule reversibly detaches from the original binding site of PDC109/a between 240 ns and 334 ns, causing divergence of the distances (left panels in Fig. 3).

Tyrosine-PhC distances in the MD simulations vary between 3.0 Å and 10.0 Å for tyrosines within the binding sites (Fig. 3 E–H), and increase up to 12 Å for tyrosines on the outer rim of the binding pockets (Fig. 3 I and J). Consistent with the crystal structure [24], the lower bound of these distances (3.0 Å) suggests H-bond formation. The MD simulations further suggest that the PhC ligand repeatedly switches between H-bonding configurations with either tyrosines at the binding site or tyrosines at the outer rim to maintain a dynamic equilibrium. These observations are also consistent with the variability seen in the crystal structure [24]. In protomer B (PDC109/a and PDC109/b domains), the PhC phosphate interacts with the tyrosines at the binding pockets. In the PDC109/b domain of protomer A, the PhC phosphate group is detached from binding site residues Y75 and Y100 and interacts closely with Y108 in the outer rim.

**Figure 4:**
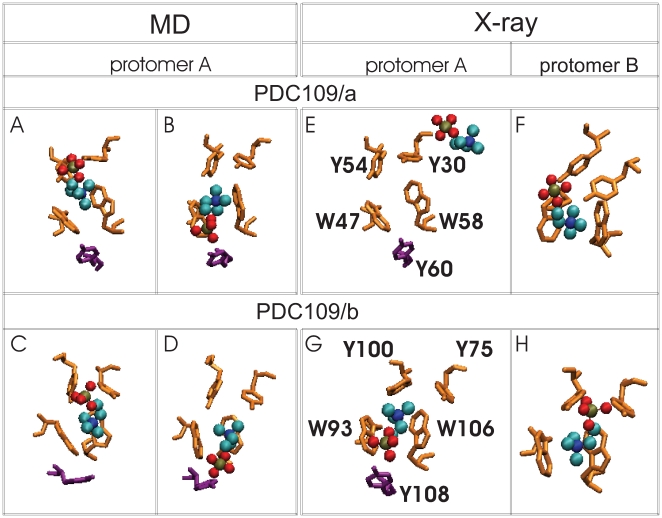
Ligand-binding site interactions. Four snapshots from the MD simulations (A
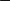
D) are compared with corresponding binding site conformations from the crystallographic protomers A and B (E
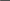
H). Snapshots showing PhC phosphate moiety interactions with specific protein side-chains are arranged as follows: (A) Y30 and Y54 at 60 ns, (B) Y60 at 100 ns, (C) Y75 and Y100 at 150 ns, and (D) Y108 at 175 ns. Binding site residues Y30, Y54, W47, W58 (from PDC109/a), and Y75, Y100, W93, W106 (from PDC109/b) are shown in orange, residues Y60 and Y108 (from PDC109/b) are shown in purple, while bound PhC molecules are shown as charge-colored spheres. Residue labels are indicated in the crystal structure panels E and G.

Fig. 4 shows a comparison between specifically chosen conformations from the MD simulations (Fig. 4 A–D) and the crystal structures (Fig. 4 E–H) of the PDC109/a (top) and PDC109/b (bottom) domains of protomers A and B [24]. This comparison illustrates ligand geometry variation in the PDC109 binding pockets. The four MD conformations show the PhC phosphate either close to tyrosines at the binding sites in Fig. 4 A and C or close to tyrosines in its outer rim in Fig. 4 B and D. As discussed above, the anomalous PhC position in PDC109/a of protomer A in the crystal structure (Fig. 4 E) is also consistent with divergence of the distances in the MD simulations (left column in Fig. 3). The primary binding site can be postulated to correspond to the original binding site in the MD simulations since in the crystal structure PhC is well posed at this site in the PDC109/b domain of protomer A and in the PDC109/a and PDC109/b domains of protomer B (Fig. 4 F–H). The different ligand geometries in the identical domains in the crystal structure protomers also suggest a degree of plasticity in the aromatic side chain arrangements at the binding site that allows conformational adaptation to ligand motions. Such “noise” in the crystal structures provides in-built error bounds for comparison with the structures obtained in the dynamic MD simulations.

#### Ligand-protein association constant

**Figure 5:**
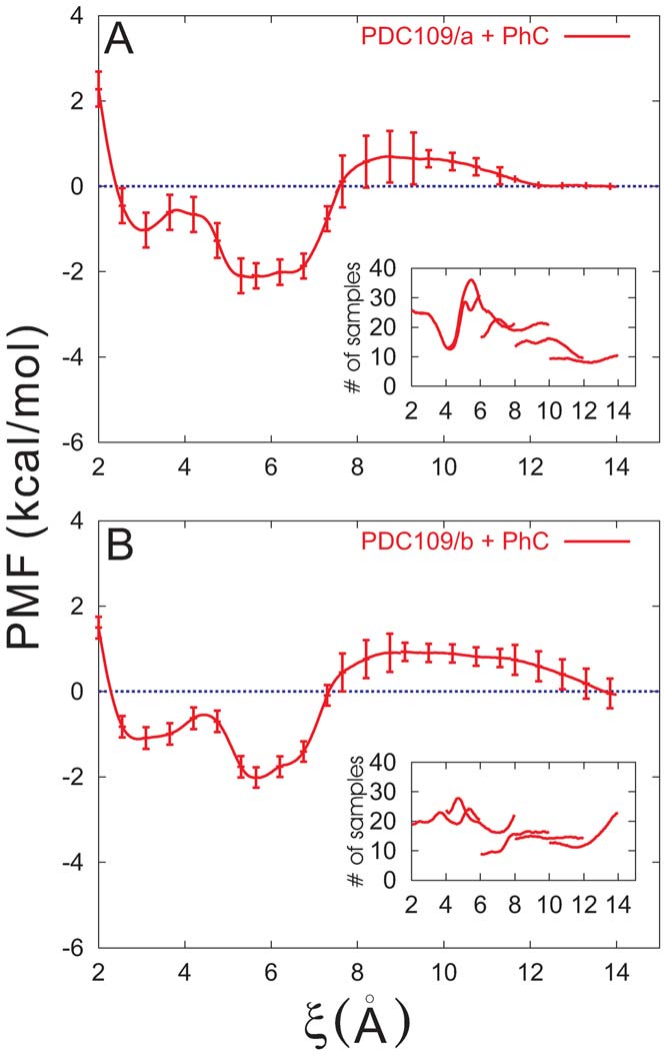
Potentials of mean force (PMFs) between PhC and the individual PDC109 domains. PMFs for PDC109/a (A) and PDC109/b (B) are displayed with standard deviations every 10 points. The insets display histogram of the number of sampling points with respect to the distance coordinate (scaled by 10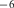).

**Figure 6:**
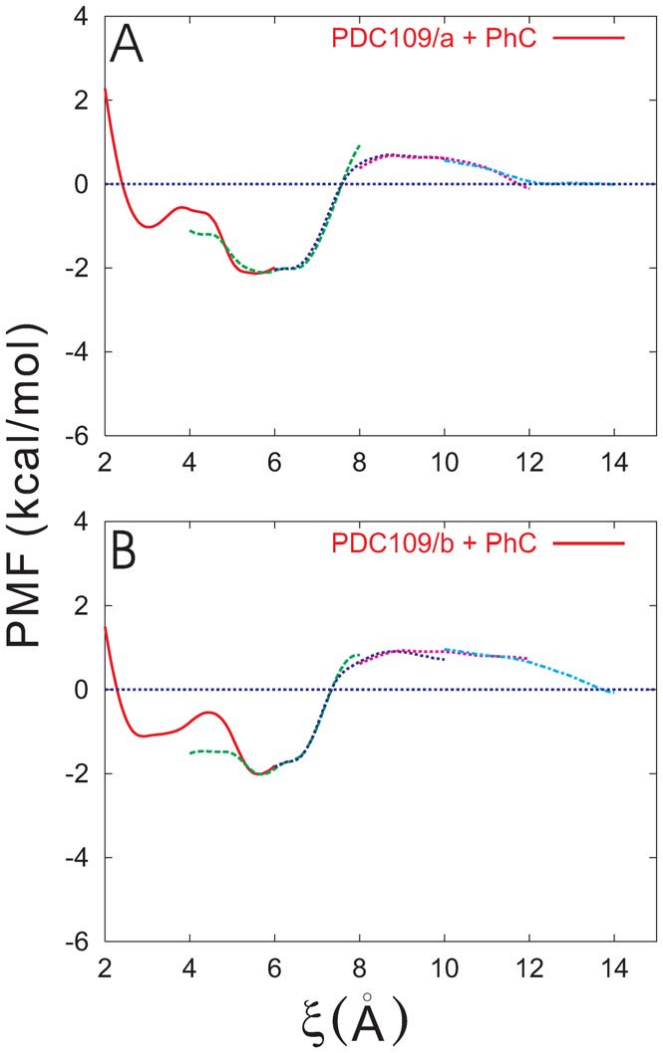
Full MD results for PMF profiles. PMFs for PDC109/a (A) and PDC109/b (B) are displayed, including those in overlapping regions of windows.

We have examined binding affinities of PhC to the PDC109 binding sites using potential of mean force (PMF) calculations. For simplicity, the distance between centers of mass of binding site atoms in a PDC109 domain and the corresponding PhC was chosen as reaction coordinate 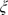. It should be noted that a reaction coordinate similar to ours is widely employed in theoretical analysis of many dissociation and association reactions in condensed phases [52]. The PMF profiles for PDC109/a-PhC and PDC109/b-PhC are displayed with their standard deviations in Fig. 5 A and B, respectively. For both domains, the PMF along 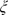 shows two local minima. In the PDC109/a-PhC case, they are located around 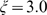 Å and 5.6 Å with respective well depths 1.0 kcal/mol and 2.1 kcal/mol. The position and well depth of the corresponding minima for PDC109/b-PhC are 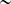
3.0 Å and 1.1 kcal/mol and 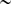
5.7 Å and 2.0 kcal/mol. In either case, the local minimum at the larger 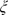 is the global minimum. The estimated errors (see Eq. 3 below) are less than 1.0 kcal/mol in all but one window with 6 Å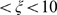 Å. In addition, Fig. 6 A and B illustrate the PMFs for PDC109/a-PhC and PDC109/b-PhC including their profiles in the overlapping regions between neighboring windows, respectively. The PMF profiles in these regions further support accuracy of the PMF calculations.

**Figure 7:**
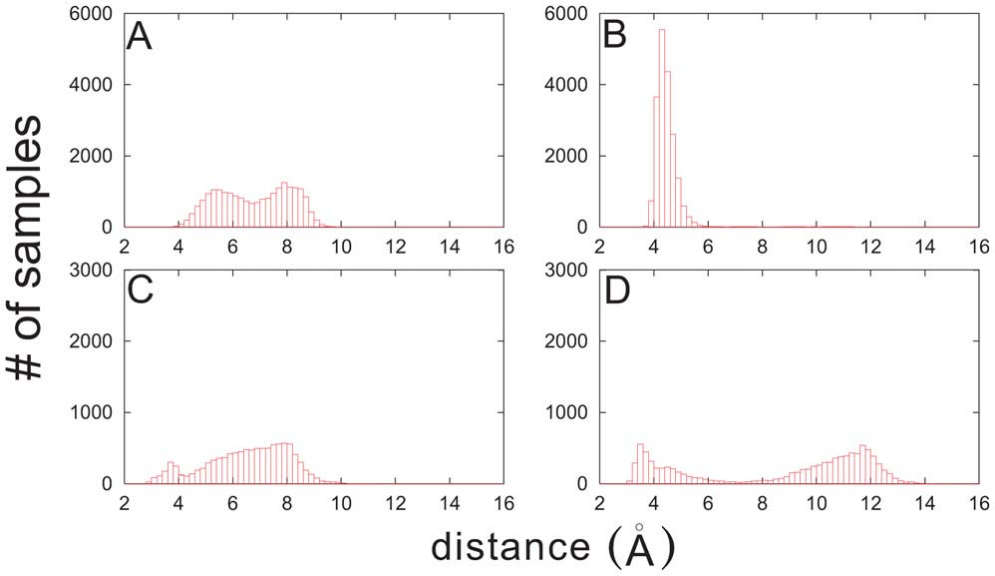
Histograms of number of samples as a function of atom-pair distances in the two minima of PMF. The distance between W47/W58 of PDC109/a and the N atom of PhC in the first (2.5 Å
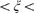
3.5 Å) (A) and second (5.2 Å
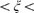
6.0 Å) (B) minima were calculated from the trajectory saved from the PMF calculation in the window 2.0 Å
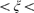
6.0 Å. The corresponding results for the separation between Y60 of PDC109/a and PhC phosphate group are shown in (C) and (D).

We have analyzed the distributions of atom-pair distances at either minimum of the PMF. Fig. 7 illustrates the results for PDC109/a-PhC. We notice that the local minimum at 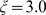 Å is characterized by a broad distribution of the separation between W47/W58 of PDC109/a and the PhC nitrogen atom (Fig. 7 A), while they form a bound structure in the global minimum around 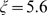 Å (Fig. 7 B). Furthermore, Y60 of the PDC109/a and PhC phosphate group show a well-defined two-state structure in the global minimum (Fig. 7 D), corresponding to H-bond formation and breaking, whereas their H-bonds are mostly absent in the local minimum near 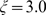 Å (Fig. 7 C). Similar trends are revealed by Y30 and Y54 (results not shown here). The distance between Y60 and PhC phosphate group in the broken H-bond state is shorter at the minimum around 3.0 Å than that at the minimum near 5.6 Å. Thus, we speculate that PhCs interact more strongly with tyrosines than with tryptophans in the local minimum, compared with the global minimum. Our analysis indicates that structures of binding sites and their fluctuations (Fig. 3) are associated with the global minimum of the PMF. This consistency between two different calculations validates the simulations. We note that the two minima of the PMF for PDC109/b-PhC are characterized by similar binding site properties (not presented here).

Values for the association constant (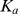) for binding of PhC to PDC109/a and PDC109/b domains were calculated using the equation [48], [53], [54]

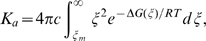
(1)where 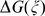 is the free energy difference between 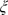 and 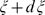 and 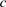, 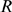, 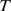 are the concentration, the gas constant, the temperature, respectively. 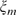 and 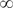 are reaction coordinate values where 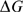 is at a minimum and zero, respectively (Fig. 5 A and B). Because of the finite 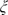 range, 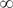 is defined as 14 Å, which would yield a lower bound for 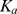. From the PMFs at 300 K, we estimate 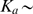
28.2 M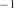 for the PDC109/a and 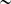
27.7 M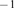 for PDC109/b, respectively. The effective 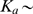
28 M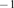 for the entire two-domain PDC109 bound to two PhC molecules was obtained by averaging the 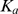 values for domains PDC109/a and PDC109/b, weighted by a Boltzmann factor. The protein concentration, 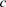, was taken as *ca.* 4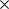 the concentration of the Fn2 domain in order to compare it with the experimental values [45] for the intact protein PDC109 dimer, namely 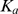 28.3 M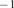 and 33.2 M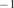 at pH 7.4, 298.15 K
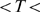
303.15 K. This indicates that the thermodynamic characteristics of PhC binding are well reproduced in our one-dimensional description despite potential uncertainties in the definition of reactant and product states with a single reaction coordinate.

### Effects of PhC Binding on Inter-Domain Dynamics

#### Relative Orientation of the two domains

**Figure 8:**
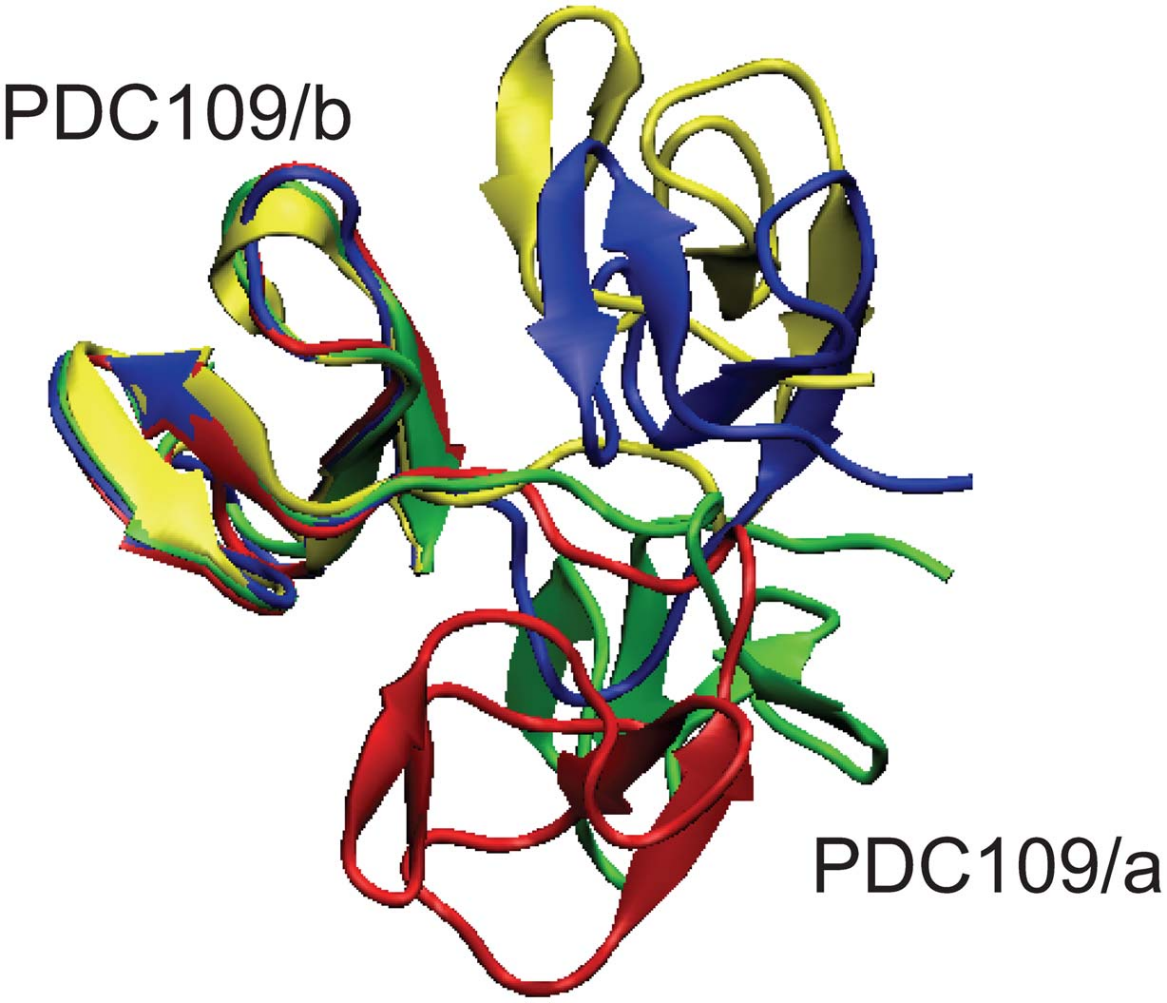
Snapshots of PDC109 during the MD simulations. Structures of the PDC109-PhC complex at 200 ns (red) and 350 ns (blue) and of ligand-free PDC109 at 350 ns (green) are compared against the X-ray crystallographic structure (yellow) via a best-fit of PDC109/b [74].

In the crystal [24], the ligand binding sites of both Fn2 domains position at the same face of the PDC109 molecules but rotated 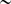
70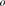 from each other. Fig. 8 compares the X-ray structure (yellow) against snapshots of the PDC109-PhC complex at 200 ns (red) and 350 ns (blue) and against a snapshot of ligand-free PDC109 at 350 ns (green) via a best-fit of the PDC109/b domain (PhC molecules are not shown). Complementing the static X-ray structure, the MD simulations reveal drastic changes in the relative orientations between the two domains at 200 ns for PDC109-PhC and at 350 ns for ligand-free PDC109, respectively. This behavior is rather similar to Fn2 domains oriented in opposite directions and faced 180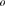 from each other in the recently reported structures of human matrix metallo-proteinase 2 (MMP-2) [55], [56]. However, in the presence of PhC, the 350 ns snapshot of PDC109 shows the relative orientation is restored and stabilized close to the X-ray structure, although the linker seems to be more compressed but slightly pushed away from the two domains.

**Figure 9:**
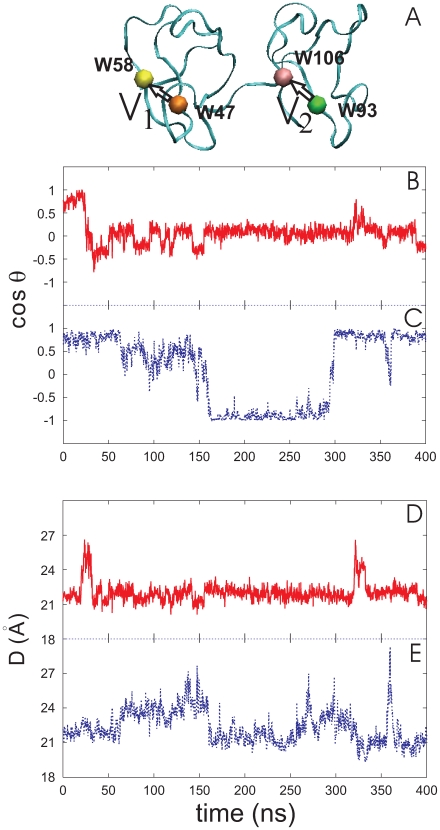
Relative orientation and distance of the two domains of PDC109. 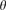
 is the angle between two vectors, **V** 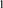 and **V** 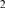 in (A) [74], defined as displacements of C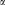 atom between W47 and W58 of PDC109/a and between W93 and W106 of PDC109/b, respectively. Fluctuations of 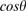 are shown for ligand-free PDC109 in red (B) and for PDC109 complexed with PhC in blue (C). Fluctuations of center-to-center distance 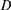 between two domains of PDC109 are in red (D) and of PhC-bound PDC109 are in blue (E).

The global variation of the relative arrangement of Fn2 domains in PDC109 was monitored by tracking 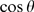, as defined by:
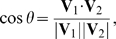
(2)where V1 and V2 represent vectors connecting the 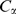 atoms of residues W47 and W58 in PDC109/a and residues W93 and W106 in PDC109/b (Fig. 9 A). These residues were chosen owing to their location within the binding site and because they exhibited, by reflecting the crystal structure, less mobility during the MD simulations. The 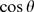 value calculated from the crystal structure is 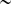
0.8, corresponding to 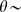
37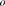. Fig. 9 B and C illustrates the time course of 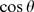 for PDC109 in the absence and presence of PhC, respectively. The 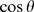 values fluctuate for both systems during the first 160 ns, with less stability seen in the values for the PhC bound protein. In the absence of ligand, the two vectors mostly remain close to perpendicular (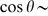
0). However, in the presence of PhC, the vectors approach anti-parallel (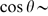
−1) orientation around 160 ns and parallel (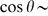
1) orientation around 300 ns. The conformational changes of the protein lead the ligand dissociation and association around 240 ns and 334 ns, respectively (Fig. 3). Interestingly, this implies that the PhC binding affinity to PDC109 varies with the relative orientation of the two domains.

The time series of the inter-domain distance, 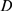, defined as the distance between the center of masses of the backbone atoms of PDC109/a and PDC109/b, for ligand-free and PhC bound PDC109 are shown in Fig. 9 D and E, respectively. In the crystal structure [24], 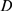 is 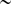
21 Å. In the absence of PhC, 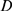 fluctuates around 22 Å, except for brief increases to 27 Å in the time ranges 20–32 ns and 320–330 ns (Fig. 9 D). In the presence of PhC, 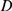 varies between 20 Å and 27 Å over the entire dynamics run (Fig. 9 E). However, once the vector orientations stabilize, 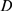 decreases to 21 Å, which is consistent with that observed from the X-ray structure of the PDC109-PhC complex. Regardless of PhC binding, 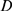 shows a rapid increase to 27 Å or more, when the two domains either initiate or undergo rapid transitions between the relative orientations.

#### Structural variation

**Figure 10:**
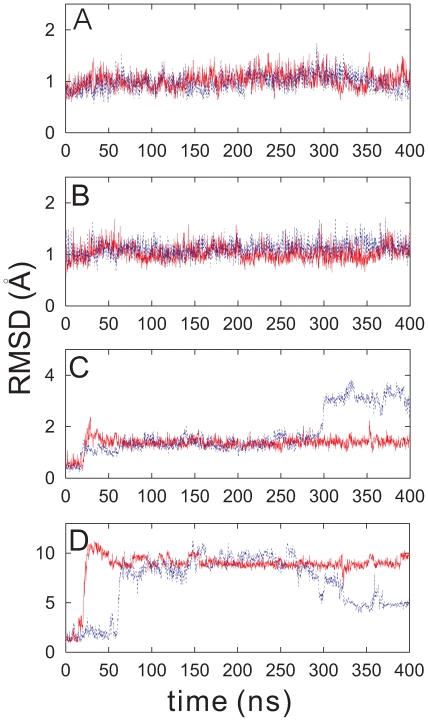
RMSDs for (A) PDC109/a, (B) PDC109/b, (C) linker, and (D) the entire protein. The time series for ligand-bound and free PDC109 is shown in blue and red, respectively.

Root Mean Square Deviation (RMSD) of the segmental backbone atoms of PDC109 was calculated relative to the crystal structure conformation of the protein complexed with PhC. As shown in Fig. 10 A and B, the RMSD values in the time series for individual domains PDC109/a (C24–C61) and PDC109/b (C69–C109) remain less than 1.5 Å, whether in the presence or absence of PhC. The long duration of the MD simulations indicates that the CHARMM force field [57], [58] is able to maintain the stability of the domains’ global folds in the absence of imposed experimental or *ad hoc* harmonic restraints. For the linker region, the RMSDs are less than 2.0 Å regardless of PhC binding, while for the PhC-bound PDC109 protein the RMSD increases and fluctuates around 3.5 Å during the last 100 ns (Fig. 10 C). This suggests that most of the conformational variability of PDC109 results from the internal conformational changes of the linker as it contracts and extends (Fig. 8). In particular, the large RMSD value of the linker segment during the last 100 ns reflects structural deformation of the linker relative to the X-ray structure (Fig. 8 blue). Fig. 10 D shows that the RMSD values markedly increase for the entire PDC109 protein, with or without bound PhC. The RMSDs increase from 2 Å to 10 Å at 20 ns for the ligand-free protein and at 60 ns for the PhC-bound PDC109. After 250 ns, the RMSD of the entire PhC-bound PDC109 gradually decreases and remains 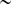
5.0 Å. This variation in the RMSD suggests that the relative orientation of the two domains drifts away from the crystal structure orientation in the absence of ligand (Fig. 8 green and Fig. 8 B); however, they can also return to the crystallographic conformation in the presence of ligand (Fig. 8 blue).

The backbone Root Mean Square Fluctuation (RMSF) of the linker are compared with those of the two domains along the MD trajectories. The RMSF value of each construct was normalized according to the number of residues. The reference structure of each segment and its standard deviation were calculated averaging over 400 ns trajectories saved every 5 ps. In the absence of PhC, the normalized RMSF of the linker is 0.066
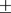
0.180, which is larger than 0.017
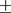
0.102 (PDC109/a) and 0.015
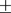
0.098 (PDC109/b). In the presence of PhC, it increases to 0.170
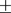
0.433, while RMSFs for PDC109/a and PDC109/b remain small, 0.016
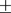
0.101 and 0.014
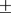
0.087, respectively. This suggests that regardless of PhC binding the most flexible region of PDC109 is the linker.

Two lines of evidence support extra flexibility for the linker segment. Tryptic digestion of the intact PDC109 cleaves the linker, at R64 thus generating two main fragments [59]. One of these contains the PDC109/b repeat which, although it carries an arginine residue at site 104, remains uncleaved. This supports linker flexibility that would enable it to be promptly digested by trypsin, while the more rigid PDC109/b module is obtained intact. Structural variation between the sequences within the two protomers in the crystallographic X-ray structure was gauged by calculating segmental RMSD (per residue) of protomer A relative to protomer B. Resulting RMSD values for PDC109/a, PDC109/b and the linker are 0.009, 0.019, and 0.030, respectively. This extra “disorder” for the linker supports a higher flexibility for the segment.

### Cooperative Motions and Intrinsic Conformational Changes

#### Normal modes and principal components

**Figure 11:**
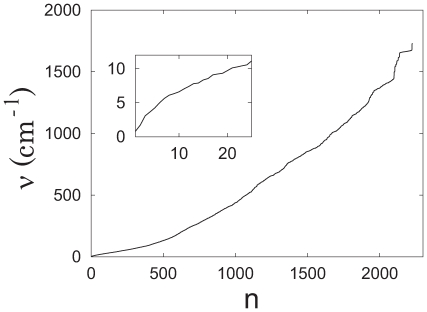
Normal mode (NM) spectrum for ligand-free PDC109 (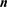 = NM index). The inset shows an expansion of the low frequency range.

The normal modes (NMs) were obtained for a minimized conformation of PDC109 from the Hessian matrix by treating individual protein atoms as harmonic oscillators in vacuum. Fig. 11 shows the computed NM spectrum of PDC109 without bound PhC ligand, where the first six NMs, corresponding to global translational and rotational motions, are ignored. The index 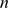 arranges NMs with non-zero frequencies according to increasing frequency values. In general, low-frequency modes correspond to global cooperative oscillations whereas high-frequency modes correspond to local oscillations. The inset in Fig. 11 illustrates the NMs with frequencies below 12 cm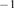.

**Figure 12:**
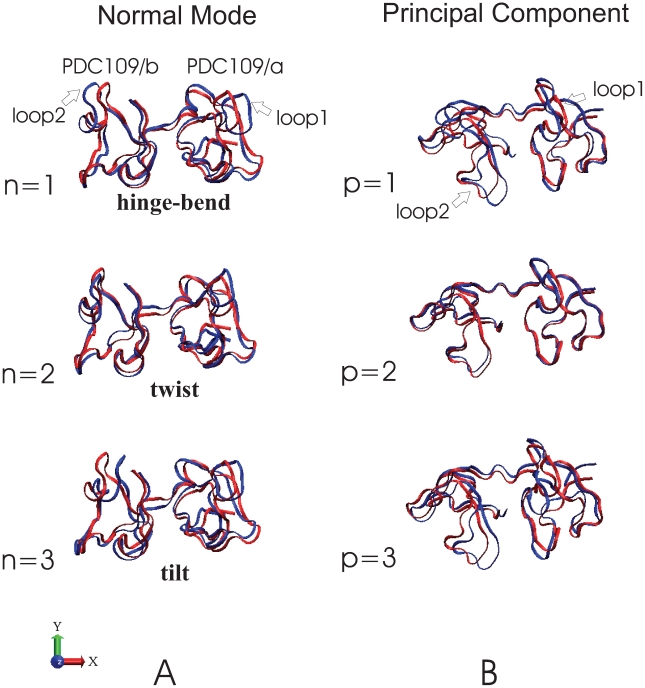
Comparison of the first three normal modes and principal components. Overlapping ribbon conformations for the three lowest normal modes are shown at t = 0 (red) and 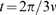 (blue) with the normalized eigenvector vibrational amplitudes scaled by a factor of 200. The normal mode index 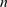 corresponds to specific vibrational frequencies as follows: 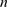 = 1 (hinge-bend), 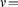
0.80 cm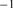; 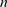 = 2 (twist), 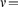
1.72 cm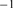; 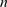 = 3 (tilt), 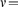
3.08 cm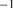. Overlapping ribbon conformations for the three largest amplitude principal components (p = 1, 2, 3) are shown with the reference structure (red) as displacements scaled by a factor of 200 standard deviations along each principal component (blue) [74].

**Figure 13:**
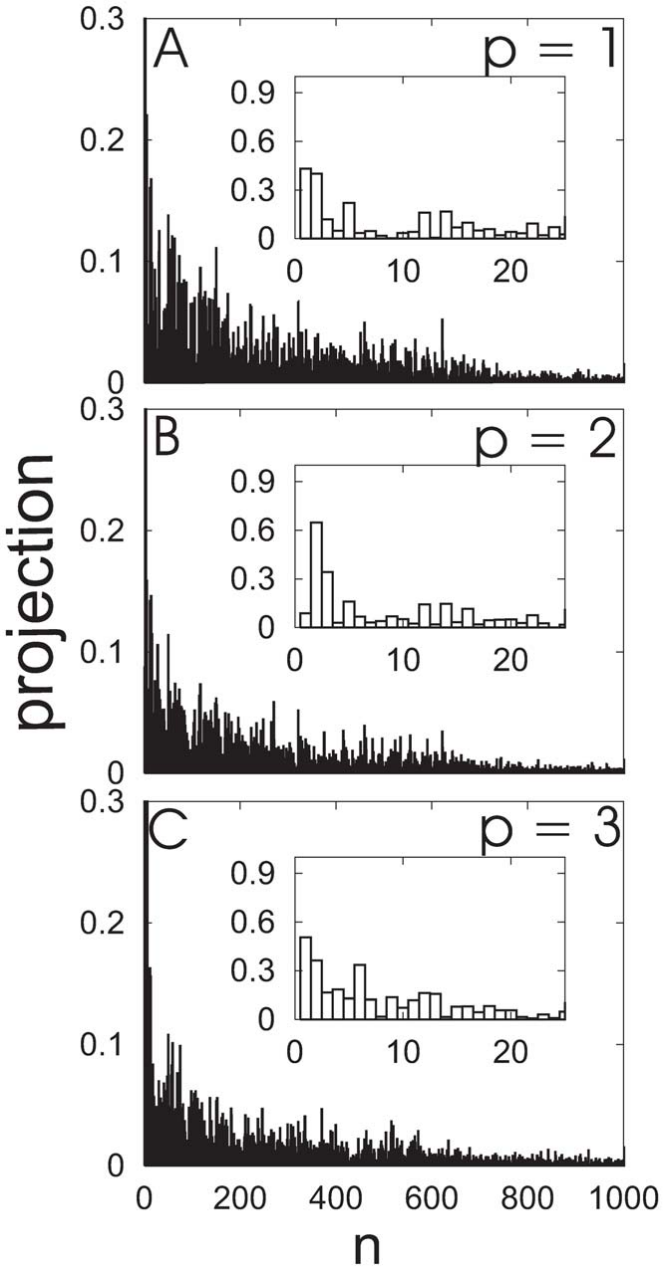
Projections of the principal components on the normal modes for PDC109. (A) p = 1, (B) p = 2, and (C) p = 3. Insets show a magnified view for low frequency modes.

Both columns of Fig. 12 show NMs and principal components (PCs) orientated via best-fits of NM and PC reference (red) against the PDC109/a structures. Fig. 12 A depicts the three lowest normal modes of PDC109, which are hinge-bend (n = 1), twist (n = 2), and tilt (n = 3) motions. The modes are shown as overlaid snapshots, where the blue snapshot is time-shifted relative to the red snapshot as described in the Fig. 12 A caption. Fig. 12 B displays the three largest eigenvalue PCs of ligand-free PDC109 as structural displacements (blue) relative to the average reference structure (red). The index 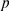 increases as the eigenvalue (or the amplitude of atomic fluctuations) decreases. PCs were calculated by diagonalizing the covariance matrix generated from long timescale (400 ns) explicit solvent MD simulations. These PCs therefore account for an anharmonic diffusion motions due to complicated solvent effects. The PC associated with the largest eigenvalue corresponds to the largest atomic fluctuations. Due to solvent damping, PCs have smaller vibrational amplitudes as compared to NMs calculated in vacuum. Compared to NM, loop 2 of each PC rotates about 180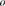 relative to loop 1. This difference stems from the average reference structure of ligand-free PDC109, obtained from the 400 ns trajectory where the two domains mostly remain in perpendicular relative orientation (Fig. 9). Each of the three largest eigenvalue PCs involves rotation of the PDC109/a domain relative to the PDC109/b domain similar to the lowest frequency NMs. To evaluate the relatedness between PCs and NMs, PCs with the three largest eigenvalues were projected onto all NMs of PDC109 as shown in Fig. 13. These projected values are displayed for n 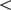
1000 with insets magnifying values for n 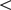
24. We find that PCs with large eigenvalues are highly correlated to low frequency NMs. The n = 1 and n = 2 NMs account for 19.1% and 16.5% of the first PC, respectively. The n = 2 and n = 3 NMs account for 37.7% and 11.9% of the second PC, respectively. Similarly, the n = 1 and n = 2 NMs account for 26.5% and 13.2% of the third PC, respectively (see insets of Fig. 13 A 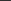
C). The contribution from the other NMs on the PCs is negligible, reflecting the fact that the amplitude of NM motion decreases rapidly as the NM index increases.

#### Correlations between cooperative changes and ligand binding

**Figure 14:**
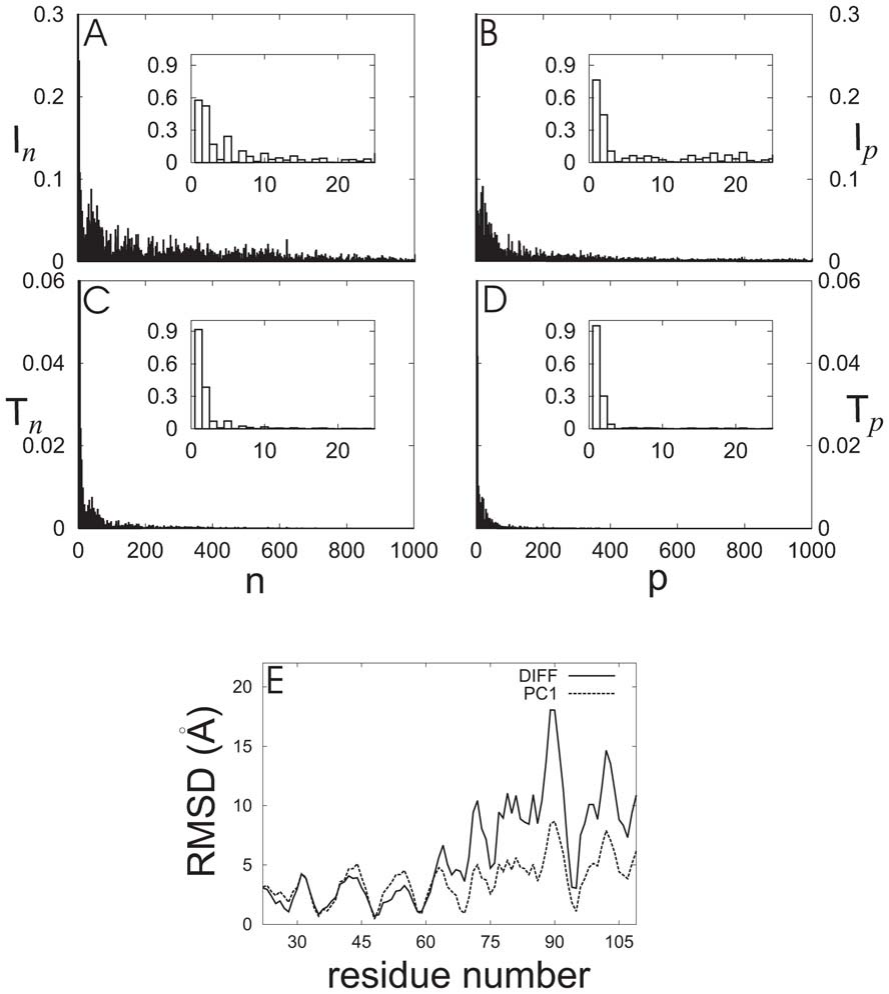
Influence of ligand binding on normal modes and principal components of PDC109. Involvement coefficients (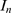) and thermal involvement coefficients (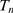) in the space spanned by normal modes 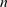 are displayed in (A) and (B), respectively. The corresponding decompositions into the principal components are shown in (C) and (D). Insets show a magnified view for low mode frequencies. (E) Residue-wise comparison of amplitudes of PC1 eigenvectors at 0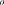 and 180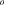 (PC1, dashed line) and conformational difference (DIFF, solid line) between ligand-free and PhC-bound PDC109. At each residue, backbone atom coordinates were averaged out and the normalized difference between opposite components was scaled by a factor of 2.36 to obtain a best-fit conformational difference.

The difference between PDC109 conformations, ligand-free and with bound PhC, was normalized by subtracting the two corresponding average PDC109 reference structures after optimal heavy atom superposition. The involvement coefficient (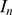) and the thermal involvement coefficient (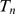 defined in Eq. 5) [60] at each NM (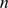), computed by using the normalized conformational difference vector (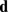) and the NM eigenvectors of PDC109, is shown in Fig. 14 A and C for n 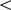
1000. The insets in Fig. 14 A and C magnify these coefficients for NMs with n 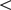
24. It can be seen from these insets that 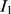, 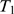, 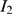 and 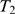 coefficients account for 28.3%, 80.3%, 29.9% and 18.1% of the distortions accompanying PhC binding, respectively; with the major contributions coming from the n = 1 and n = 2 NMs.

The corresponding involvement coefficients (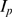) and (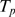) at each PC (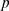) are shown in Fig. 14 B and D. As seen in the insets, 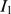 and 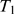 account for 57.7% and 90.6% of the overlap between the average conformational difference for PDC109 (ligand-free and with bound PhC) and the first PC. For higher PCs, 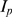 and 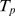 contributions become much smaller. This suggests that the average conformational differences between PDC109 with and without bound PhC mainly are related to the first PC. Finally, Fig. 14 E shows the residue-wise decomposition of the average conformational differences and the eigenvalue amplitude of the first PC1 (adjusted to minimize the RMSD between the PC1 eigenvector and the conformational difference vector, 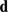). The close agreement between the two curves in Fig. 14 E also reflects the observation that the first PC can account for most of the conformational changes associated with PhC binding to PDC109.

### Conclusions

PDC109, a modular two-domain protein, is the most abundant protein in bull seminal plasma and is involved in sperm capacitation by interacting with sperm cell membranes [25]. Many experimental studies have reported that the interactions of PDC109 with phospholipid membranes induce the specific binding of each PDC109 domain with PhC, head group of choline phospholipids [24]–[46]. In order to better understand their interactions, we have investigated effect of PhC binding on global as well as local conformational changes in the two-domain protein, PDC109 as a model system. To the best of our knowledge, this is the first MD study on role of ligand binding on dynamic motions of the multi-domain protein.

Our MD simulations suggest that when bound to PhC the two domains of PDC109 undergo transitions between the parallel and anti-parallel relative orientations. The transitions result in dissociation and association of PhC to PDC109/a around 240 ns and around 334 ns, respectively, emphasizing the dynamic nature of the PhC binding to its binding site. When PhC rebinds to PDC109/a, the two domains restored the parallel orientation, which is close to the relative orientation in the X-ray crystallographic structure, but allowing deformation of the linker. Compared against the crystallographic structure, our model may suggest a more stable structure of the PhC-bound PDC109. However, in the absence of ligand, the two domains favor the perpendicular relative orientation. This implies that the relative domain motions of PDC109 can drastically influence its association with ligand. Note that during the conformational changes, the structure of each domain remains rather rigid in contrast with the flexible linker, which affords a hinge for the angular rearrangements.

It has been shown [44] that in solution, PDC109 exists as a polydisperse aggregate, mixture of monomer and oligomers, which upon PhC binding dissociates into dimers, as found in the crystal of the PDC109-PhC complex [24]. This suggests that when one of the two domains in each monomer binds the PhC head group of the bull spermatozoa membrane phospholipids, binding of the second domain to a neighboring PhC head group is likely to involve reorientation of the second domains relative to the first one, such that the two domains end up pointing in the same direction, in a parallel configuration. This may be of biological relevance and would be consistent with the structures of PDC109 we obtain via MD, being compatible with a hypothetical unbound monomer and membrane bound dimer configurations.

There have been many studies of protein conformational changes via NMA and PCA [47]. However, our study on PDC109 would be a first instance in which these methods have been applied to the analysis of domain motions in two-domain protein. The three lowest NMs obtained from NMA and the first PC obtained from the PCA account for most of the conformational difference between ligand-free PDC109 and the PDC109-PhC complex seen in the long (400 ns) timescale MD simulations. This finding suggests that the NMA method may be employed without sacrificing much accuracy to probe conformational characteristics of multi-domain protein at a significantly low computational cost.

The interactions of the binding site tryptophans with the ligand in the MD simulations mostly occur within the range of distances revealed by crystal structure, which suggests that there is no specific hindrance for these cation-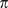 type interactions in explicit solvent MD simulations. In contrast, PhC interactions with the binding site tyrosines seem to stochastically form and break. This erratic behavior for the H-bonds involved occurs at higher frequency in PDC109/b than in PDC109/a. This is consistent with the observed variability of interaction of PhC with the two different sets of tyrosine residues in the crystal structure and suggests that the electrostatic interactions between PhC and the binding site tyrosines are much more susceptible to be maintained, while in a dynamic equilibrium.

Our PMF calculation for each domain-PhC complex indicates that binding site structures and their fluctuations are associated with the global minimum of the PMF. The ligand-PDC109 association constant obtained using PMF calculations, 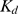 = 28M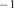, is also consistent with the experimental values [45]. This indicates that the underlying energy function used in the MD simulations is of sufficient accuracy to model the local conformational dynamics.

It is our hope that the present molecular dynamics characterization of PDC109 helps provide insights into the effects of ligand binding of relative domain motions in a multi-domain protein. It also provides a framework for future efforts to understanding flexibility of more complicated macromolecular complexes and their bindings (or interactions) with cellular membrane components.

## Methods

The homodimer crystal structure of PDC109 complexed with PhCs was obtained from the Protein Data Bank (PDB ID: 1h8p).

### Initial Structural Model

In the crystal structure [24], the 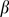-strand segments R32
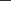
H34, W47
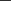
S49 in PDC109/a and K78
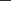
Y80, W93
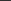
S95 in PDC109/b form short 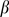-sheets, resulting in a homodimer interface (Figs. 1 and 2). Segments between the two 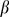-strands, H41
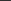
L44 (PDC109/a) and G87
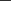
M91 (PDC109/b), are called 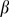
2–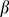
3 (loop1) and 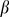
2
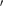
–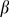
3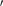 (loop2) [24]. Since BSP-A1 (protomer A) and BSP-A2 (protomer B) have similar structures [24], we chose the BSP-A1 monomeric unit (Fig. 2 A) for our study. All simulations were carried out using the NAMD program [61] with the CHARMM27 [57], [58] force field including the CMAP backbone potential. Hydrogen atoms were added to the X-ray structure (Fig. 2 A) of PDC109 bound to two PhC ligands using CHARMM (v31b2) with histidine sidechains protonated at their N atoms. The ligand-free PDC109 structures were generated by removing the two PhC molecules. The net charges of PDC109 and PhC were 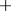
4 and 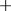
1, respectively. Atomic electrostatic charges for the PhC ligand were built following bond increment rules using the Momany and Rone CHARMm parameterization method [62], [63]. The added H-atoms were minimized using the steepest-descent (SD) algorithm with harmonic constraints on the heavy atoms, whose force constants were gradually decreased from 30 kcal/mol/Å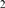 to 0 kcal/mol/Å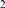 during the 1500 step SD minimization. The resulting structure of ligand-free PDC109 was solvated in explicit solvent without additional optimization.

The crystal structure of PDC109/a bound to PhC [24] shows that the quaternary ammonium of PhC interacts with the buried W47 indole and the phosphate of PhC hydrogen bonds with Y30 and Y54 in PDC109/a. In PDC109/b, the PhC quaternary ammonium group interacts with the exposed W106 rather than the buried W93 ring and the PhC phosphate H-bonds with Y75 and Y100 hydroxyl groups. While in the crystal structure the PhC molecule in PDC109/b is located outside the binding pocket (Fig. 2 A), it was placed inside the pocket during the preparation of the structural model for the MD simulations to maintain symmetry in the two domains. In order to restrict the domains to remain close to their initial folds, harmonic constraints with a force constant of 100 kcal/mol/Å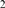 were applied to all heavy atoms. For PDC109 complexed with two PhC molecules, harmonic constraints with force constant 20 kcal/mol/Å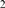 and minimum at 3.0 Å were imposed to keep the ligand stable with the respective binding pockets. For PDC109/a, these constraints were for distances between the quaternary ammonium nitrogen of PhC and the center of geometry of the six indole ring carbons of W47 and W58, and for the distance between the center of geometry of the three phosphate oxygens of PhC and the hydroxyl oxygens of Y30 and Y54. For PDC109/b, the distance constraints were with W93 and W106, and Y75 and Y100, respectively. A 1500 step SD energy minimization yielded the constrained distances to range between 3.5 Å and 5.3 Å (Table 1). Heavy atom RMSDs were 0.03 Å and 0.04 Å for domains PDC109/a and PDC109/b, respectively.

**Table 1:** MD and X-ray results for distances between binding sites of PDC109 and PhC.

		MD	Minimized	X-ray 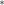	
domain	residue	protomer A	protomer A	protomer A	protomer B
PDC109/a	Y30	6.6 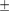 2.2	3.9	7.1	3.8
	Y54	6.9 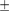 2.1	4.3	12.4	3.8
	W47	4.6 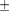 0.7	4.5	13.3	4.2
	W58	4.6 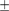 0.7	4.7	16.7	4.4
	Y60	8.1 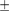 3.1	6.8	14.0	10.6
	Y75	5.9 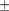 2.0	5.3	9.3	3.9
PDC109/b	Y100	6.1 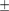 1.9	3.5	8.4	3.9
	W93	4.6 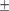 0.8	4.0	4.2	4.3
	W106	4.5 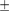 0.6	5.0	4.9	4.2
	Y108	8.8 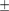 2.6	7.3	3.5	10.4

Distances are in Å and were determined by averaging through MD trajectories (protomer A) and from the crystal structure (protomers A and B, *PDB ID: 1h8p). Distances for tryptophans were calculated between the six-carbon ring centers of sidechain indoles and the PhC quaternary ammonium nitrogen. Distances for tyrosines were calculated between sidechain hydroxyl oxygens and the average position of three PhC phosphate oxygens. Average MD distances and standard deviations were calculated using the initial 230 ns trajectories because PhC started to detach from the binding pocket of PDC109/a at about 240 ns.

### Equilibration and Dynamics

The minimized protein structures for ligand-free and PhC-bound were placed in an orthorhombic box with dimensions 100 Å
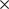
100 Å
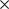
100 Å containing 14,050 and 15,017 TIP3P [64] water molecules, respectively, using SOLVATE1.0 [65]. Electroneutrality was achieved at a salt concentration of 150 mM by adding 37 Na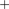 and 41 Cl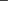 ions for PDC109 and 39 Na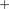 and 45 Cl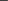 ions for PDC109 complexed with two PhC molecules. Long-range electrostatic interactions were computed via the particle-mesh Ewald method [66] with an r-space cutoff of 12.0 Å. The same cutoff was applied to the Lennard-Jones (LJ) interactions. Covalent bonds involving hydrogens were constrained using SHAKE [67].

The solvated systems were equilibrated in several stages. In order to remove initial bad contacts, energy minimization was performed using the SD algorithm. The structures of ligand-free and PhC-bound PDC109 were frozen initially for 1000 steps, then allowed to move under harmonic constraints with force constant of 100 kcal/mol/Å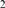 for 10000 steps. Additional minimization was performed using the SD and conjugate gradient (CG) algorithms for 6000 steps. After minimization, the temperature was gradually increased in 10 ps from 0 K to 300 K (6 K every 0.2 ps) with the protein atoms subject to harmonic constraints, followed by Langevin dynamics for 1 ns, during which the harmonic constraints were gradually lifted. The systems were then equilibrated without constraints for 5 ns via the Langevin piston Nosé-Hoover method [68], [69]. A timestep was set to 2 fs. During the equilibration, the periodic boxes reduced in volume to the dimensions 76.5 Å
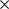
75.0 Å
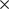
75.0 Å for PDC109 and 78.3 Å
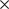
76.7 Å
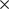
76.7 Å for PDC109 complexed with two PhC molecules. Finally, production dynamic runs were performed for 400 ns with trajectories saved every 2 ps.

### PMF Calculations

The PMF to estimate binding affinity of PhC to each PDC109 domain was carried out using the adaptive biasing force (ABF) method [70], [72]. Each ligand interaction with individual PDC109 domains was calculated separately to minimize computational time under the assumption that PhC interaction with each of the two domains is uncorrelated with the other domain. Counter-ions and water molecules located more than 30 Å away from the protein boundary were removed from the equilibrated structure of each domain bound to PhC. This reduced the solvent box to 19 Na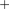, 21 Cl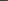 ions and 9681 TIP3P water molecules for PDC109/a bound to PhC and to 20 Na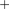, 23 Cl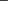 ions and 9712 water molecules for PDC109/b bound to PhC. An equilibration procedure similar to that used for the unrestrained MD simulations was applied. It involved 15000 steps of energy minimization, followed by 10 ps heating and 500 ps equilibration dynamics with gradually weakening constraints.

For convenience, we employed the distance between centers of mass of specific groups of atoms at each domain’s binding site (six indole carbons of tryptophans or hydroxyl oxygens of tyrosines) and the PhC ligand (quaternary ammonium nitrogen or three phosphate anionic oxygens) as a reaction coordinate (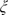) for the PMF calculations. Specifically, for binding of PhC to PDC109/a, 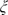 was the distance between centers of mass of the atomic groups of PhC and of Y30, Y54, W47, and W58. For PDC109/b-PhC, the corresponding distance between PhC and Y75, Y100, W93, and W106 was employed as 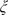. We studied the PMF for 2 Å
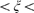
14 Å. For each domain, free energy calculations were performed in five separate windows, each 4 Å wide along 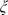 with a 2 Å overlap with its two neighboring windows. Harmonic restraints with a force constant 100 kcal/mol/Å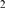 at the edges of the window were imposed to keep the solute in the prescribed region. ABF simulations in each window were conducted for 170–200 ns in the range 2 Å
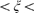
10 Å and for 85–130 ns for 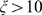 Å. The average forces were accumulated in 0.05 Å wide bins. A timestep of 1.0 fs was used to integrate the equations of motion.

The accuracy of PMF calculation was estimated by employing the degree of overlap of PMF profiles between the neighboring windows. To be specific, the error SD[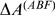] [71], [72] was determined as the standard deviation of the free energy difference, 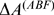, between points 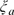 and 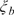


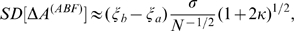
(3)where 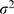 is the variance of thermodynamic force along 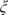, 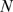 is the total number of force values computed in the entire simulation, and 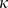 is the correlation length [73] for the series of calculated forces.

MD simulations were carried out using 128 processors in parallel using the Cray XT3 bigben cluster at the Pittsburgh Supercomputing Center.

### Normal Mode Analysis

For the normal mode analysis (NMA), we minimized PDC109 and PDC109 complexed to two PhC molecules with a 16 Å cutoff for nonbonded interactions. All hydrogens were excluded from the calculations to decrease the number of degrees of freedom and their electrostatic charges were absorbed into those of the attached heavy atoms. Calculations for PDC109 and PDC109 bound to PhC consisted of 744 and 766 heavy atoms, respectively. In order to decrease structural changes during the minimization, harmonic constraints were applied on all heavy atoms with a force constant of 100 kcal/mol/Å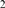 and decreased by 20 kcal/mol/Å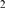 at each minimization step until complete removal of the constraints. The SD method was employed initially; once the energy gradient RMSD decreased below 10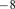 kcal/mol/Å the adopted basis Newton-Raphson (ABNR) algorithm was used to further reduce it to 10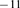 kcal/mol/Å. This magnitude of the energy gradient is expected to be sufficient for the determination of normal modes [60]. Normal modes (NMs) and vibrational frequencies were calculated from the minimized protein structures via diagonalization of the mass-weighted second-derivative matrix, using the VIBRAN module of CHARMM.

Principal components (PCs) were also examined by diagonalizing the mass-weighted coordinate covariance matrix after removing net translational and rotational motions relative to the minimized protein structures. For each solute, the structure employed as the reference structure for the covariance matrix calculation was the one averaged over the final 30 ns of its MD trajectory. We then compared PCs and NMs by projecting each PC vector onto NM vectors.

The normalized vector 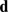, that accounts for the conformational change between the free and ligand-bound structures of PDC109, was examined:
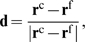
(4)where 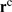 and 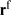 are locations of N heavy atoms of PDC109 in the 3N
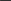
6 dimensional space for the reference structures of the PhC-bound and free PDC109, respectively. In order to obtain the overlap between the 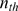 NM eigenvector, 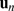, and the binding-induced deformation, the involvement coefficient [60] I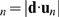, was calculated. The mode-specific involvement coefficient I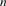 gauges the geometric similarity between 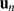 and the deformation 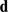. High frequency modes usually do not contribute significantly to large-amplitude motions as their amplitudes decrease as 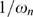 where 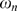 is the 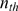 NM angular frequency. In order to assess the contribution of 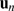 to the motion of the protein, we considered the thermal involvement coefficient [60]

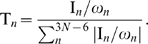
(5)The involvement coefficient and the thermal involvement coefficients, I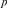 and T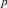, for the 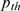 principal component were calculated in a similar fashion.
